# 

**Published:** 1831-03

**Authors:** 


### MONTHLY LIST OF MEDICAL BOOKS.

[*Medical Works cannot be entered on this List unless a copy be sent for the purpose, the titles of Books having frequently been sent to us as published, which have not been published for weeks, or even months, after.*]

Lecture, Introductory to the Course of Medical Jurisprudence, delivered in the University of London, on Friday, January 17, 1831. By A. T. Thomson, M.D. F.L.S. &c. London: Taylor.

Medical Jurisprudence has at length taken the rank in the professional education of this country, to which its vast importance so justly entitles it. If any student should be inclined to pass over this interesting and necessary branch of his studies, we recommend him to read this very eloquent lecture, which must convince him that he cannot neglect it, without great injury to himself, and injustice to the public. The Society of Apothecaries now, indeed, require a course of Medical Jurisprudence, as a part of the qualifications of a candidate for a licence to practise as an apothecary in England and Wales. The course of lectures now delivering at the London University upon this subject, are given by Dr. A. T. Thomson, and a gentleman of high reputation in the law, Professor Amos.

A Treatise on Pathological Anatomy; by G. Andral, Professor to the Faculty of Medicine of Paris, Member of the Royal Academy of Medicine, of the Council of Health, &c. Translated from the French, by Richard Townsend, A.B. M.D. M.R.I.A., and Wm. West, A.M. M.D. M.R.I.A. Vol. II. —8vo. pp. 808. Longman, London; Hodges and Smith, Dublin, 1831.

Explanation of the Anatomical Atlas of Dr. M. J. Weber, Professor at the Royal Prussian University, Frederick William, at Bonn.—London: Schloss, Southampton buildings, 1831.

This work consists of eleven well-executed plates, representing the principal parts of the anatomy of the human body, together with a copious descriptive text. Teachers of anatomy will find them of material assistance in their lectures, and students cannot fail to derive great advantage from studying them.

An Introduction to the Study of Human Anatomy. By James Paxton, Surgeon, Honorary Member of the Ashmolean Society, Author of the Notes and Illustrations of Dr. Paley's Natural Theology, &c. Vol. I.; with numerous Illustrations.—8vo. pp. 415. Vincent, Oxford, 1831.

For our opinion of this work, we refer to the Review department of the present number.

An Introduction to Medical Botany; comprehending the Elements and Terminology of Botany; the Linnæan Artificial and Natural Systems; a comprehensive Arrangement of Medical Plants; and a Table of English and Linnæan Names. Illustrated with coloured Figures. An improved Edition, containing an Arrangement of the Medical Genera according to Jussieu; and an Analytical Table of the Properties of Medical Plants. By Thos. Castles, F.L.S. of Queen's College, Oxford; Professor of Materia Medica to the Eclectic Society, &c.—18mo. pp. 202. Cox, London, 1831.

Physiology of the Fœtus, Liver, and Spleen. By George Calvert Holland, M.D., Bachelor of Letters of the University of Paris; formerly Senior President of the Hunterian Medical Society, and President of the Royal Physical Society of Edinburgh; Lecturer on Physiology, and Joint Lecturer on the Practice of Physic in the Sheffield Medical Institution.—8vo. pp. 262. Longman, London, 1831.

### NOTICES.

*Communications have been received from* Mr. James Wallace, Dr. Barry, Mr. Mitchell, Mr. Gilbert Burnett, Mr. Fraser, Mr. Ashford, &c.

*The* Editor *is much gratified by the polite note from the* Editors of the Glasgow Medical Journal. *The offer of exchanging Journals has been forwarded to the Publisher.*

*The* Editor *is unwilling to entangle himself in the disputes relating to the Nottingham Dispensary. The "exclusion" complained of appears to have arisen, not from any* general *dislike or objection to the medical profession, but from some personal misunderstanding, or difference of opinion, between the parties concerned.*

